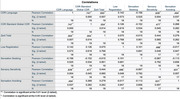# Communication Impairment in Adults with Alzheimer's Disease and Related Dementias Associated with Elevated Caregiver Burden and Sensory Processing Abnormalities

**DOI:** 10.1002/alz70858_105215

**Published:** 2025-12-26

**Authors:** Nancy L. Wolff, Clarissa I. Benzarti, Laura Henley, April L. Stauffer, Brian G. Carter, Elizabeth K. Rhodus

**Affiliations:** ^1^ University of Kentucky, Lexington, KY, USA; ^2^ HealthPRO Heritage, Louisville, KY, USA; ^3^ Sanders‐Brown Center on Aging, Lexington, KY, USA; ^4^ University of Kentucky College of Medicine Department of Behavioral Science, Lexington, KY, USA; ^5^ University of Kentucky College of Medicine Sanders‐Brown Center on Aging, Lexington, KY, USA

## Abstract

**Background:**

Declining communication is one of the hallmark symptoms of Alzheimer's disease and related dementias (ADRD). Not only is this symptom distressing for the person with ADRD, but care tasks may be more challenging as a result of communication deficits. While it is well known that hearing loss is strongly related to communication capacity, a lesser explored area related to communication is that of cortical‐level sensory processing in ADRD. We hypothesized communication impairment would be positively correlated with caregiver burden and sensory processing abnormalities in older adults with ADRD.

**Method:**

Using data collected from a non‐pharmacological randomized controlled trial aimed at behavior modification in ADRD (participants with ADRD confirmed by Clinical Dementia Rating Scale [CDR] score of 1+ and their primary caregivers), this study conducted secondary data analysis using Pearson correlation to assess relationships among communication impairment as indicated on the CDR, caregiver burden (as measured by the Zarit Burden Inventory), and sensory processing abnormalities (as measured by the Adult Sensory Profile). Demographic data were assessed using summary statistical assessment.

**Result:**

Data were analyzed from 19 participants with ADRD. Participants with ADRD consisted of 11 females, 8 males, x̄ age of 78.21 (SD=10.15), and x̄ Standard Global CDR of 1.63 (SD=0.84). All participants had functioning sensory acuity with or without aids (i.e., hearing aids). Care partners consisted of 16 females, 3 males, x̄ age of 62.32 (SD=11.56), and spouses were the most frequent care partners. Analyses indicated a significant strong positive correlation between communication impairment and caregiver burden (*r* = 0.59, *p* = 0.007). Additionally, communication impairment was significantly positively correlated with sensory processing abnormalities within the domains of sensory sensitivity (*r* = 0.64, *p* = 0.004) and sensory avoiding (*r* = 0.49, *p* = 0.037).

**Conclusion:**

Both receptive (hearing) and expressive (talking) communication is vital to encourage cooperative completion of care tasks. Communication impairment in ADRD is not only linked to increased caregiver burden, but also is associated with cortical‐level sensory processing abnormalities beyond standard hearing loss. Combined with hearing loss, communication is made even more difficult. Additional exploration is warranted to determine causal mechanisms between sensory processing abnormalities and communication impairment in ADRD.